# Assessment of the biological effect of metal ions and their complexes using *Allium cepa* and *Artemia salina* assays: a possible environmental implementation of biological inorganic chemistry

**DOI:** 10.1007/s00775-022-01963-2

**Published:** 2022-09-23

**Authors:** Chrysoula S. Tzima, Christina N. Banti, Sotiris K. Hadjikakou

**Affiliations:** 1 Section of Inorganic and Analytical Chemistry, Department of Chemistry, University of Ioannina, 45110 Ioannina, Greece; 2 Institute of Materials Science and Computing, University Research Center of Ioannina (URCI), Ioannina, Greece

**Keywords:** Environmental biological inorganic chemistry, *Allium cepa*, *Artemia salina*, Metal complexes, Metal ions

## Abstract

The pollution of aquatic ecosystems due to the elevated concentration of a variety of contaminants, such as metal ions, poses a threat to humankind, as these ecosystems are in high relevance with human activities and survivability. The exposure in heavy metal ions is responsible for many severe chronic and pathogenic diseases and some types of cancer as well. Metal ions of the groups 11 (Cu, Ag, Au), 12 (Zn, Cd, Hg), 14 (Sn, Pb) and 15 (Sb, Bi) highly interfere with proteins leading to DNA damage and oxidative stress. While, the detection of these contaminants is mainly based on physicochemical analysis, the chemical determination, however, is deemed ineffective in some cases because of their complex nature. The development of biological models for the evaluation of the presence of metal ions is an attractive solution, which provides more insights regarding their effects. The present work critically reviews the reports published regarding the toxicity assessment of heavy metal ions through *Allium cepa* and *Artemia salina* assays. The in vivo toxicity of the agents is not only dose depended, but it is also strongly affected by their ligand type. However, there is no comprehensive study which compares the biological effect of chemical agents against *Allium cepa* and *Artemia salina*. Reports that include metal ions and complexes interaction with either *Allium cepa* or *Artemia salina* bio-indicators are included in the review.

**Graphical abstract:**

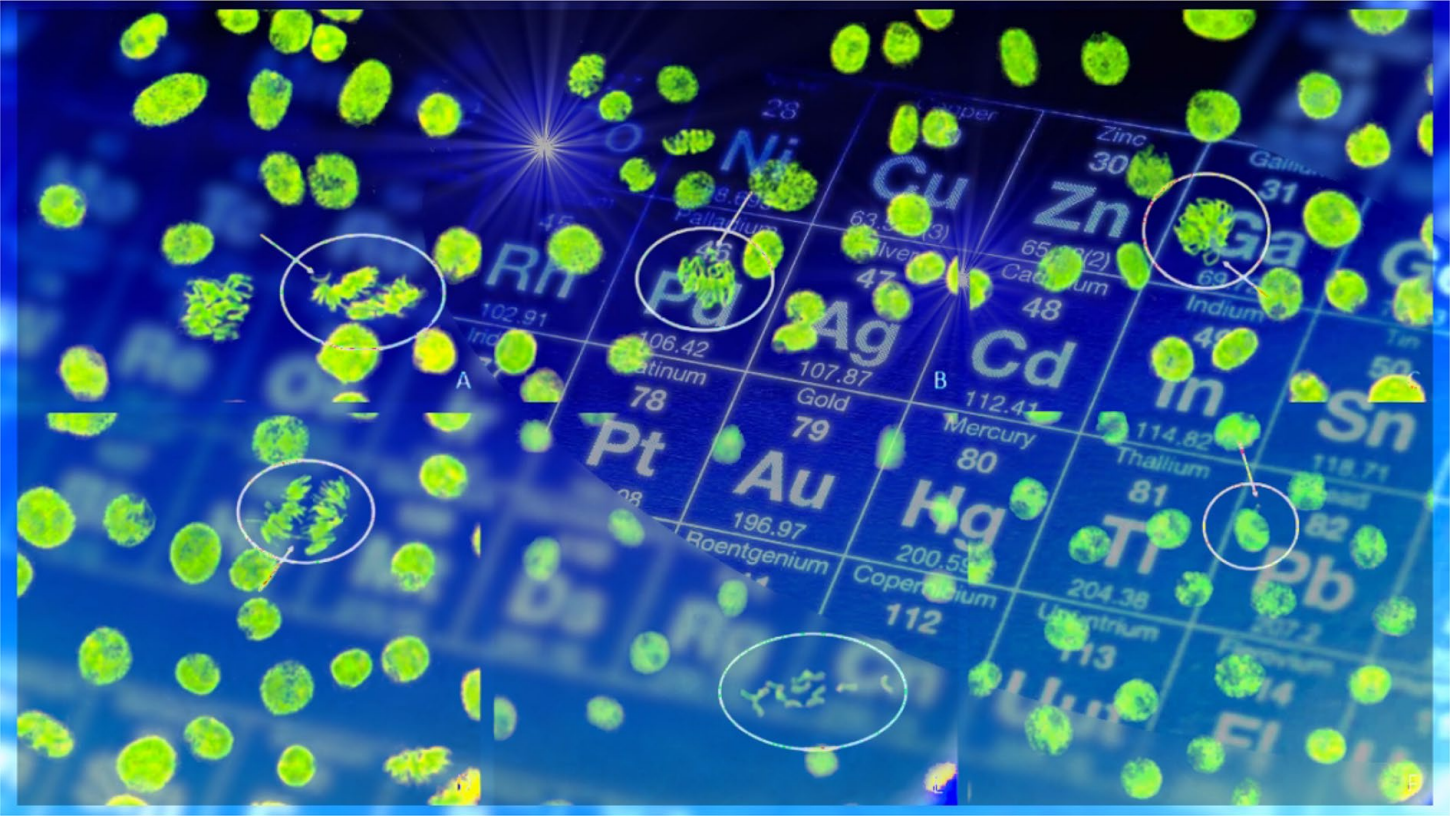

## Introduction

Although some metal trace elements are essential for life, playing an important role e.g., in transportation and signaling between cells, however, metal ions, such as Cd, Pb, As, Cr and Hg, is considered as hazardous to the health even at low concentration [1, 2]. The toxicity of heavy metals is emerged from their ability to inhibit enzymes, cause oxidative stress and suppress the antioxidant mechanisms, leading to DNA damage [2]. Moreover, the heavy metals impair the function of the nervous system causing Alzheimer’s disease and neuronal disorders [1]. Chronic inflammatory diseases and cancer are some of the most well-known pathogenic effects of heavy metals in human [2]. Ni and its compounds may cause respiratory cancer, inhalation disorders, dermatitis and reproductive problems [3]. Extended exposure to Ni leads to genotoxic and epigenetic changes, rendering Ni a possible carcinogenic agent [3]. Pb mainly induces oxidative stress and renin–angiotensin system stimulation [1]. It may disrupt the normal regulation of heart’s autonomic nerve, provoking many heart diseases, such as hypertension, coronary heart disease, stroke and peripheral arterial disease [1]. In addition, its presence has been linked with erythropoiesis and heme biosynthesis problems, anemia and some cancer types [1]. Cd is also carcinogenic and affects kidneys, bone metabolism and reproductive and endocrine systems [1]. Cd’s ability to activate calmodulin results in muscle dysfunctions and diseases like Itai-Itai disease and renal tubular dysfunction [1]. Moreover, Hg binds to enzymes and proteins, causing pneumonitis, non-cardiogenic pulmonary edema and acute respiratory distress [1]. It is considered to be an extremely hazardous element, because of its ability to cross the blood–brain barrier [1]. Methylmercury is a known neurotoxin [1]. Minamata disease is one of the diseases caused by Hg [1].

Humans are exposed to heavy metals mainly through food, cosmetic products, automobiles, radiation and effluents from a variety of industries [4]. The effort to restrict the exposure, the intake and the absorption of heavy metals by humans led the World Health Organization (WHO), Food and Agriculture Organization of the United Nations (FAO) and European Union (EU) to the establishment of guidelines regarding their concentration in food [5], drinking water [6] and water for irrigation purposes [7]. Especially the contamination of the environment due to heavy metals is a severe problem with which humankind has to deal [8]. Thus, the monitoring and the assessment of heavy metals in ecosystems is considered essential to manage the pollution they cause [8]. Since complexes formation of metal ions with ligands change the metal adsorption, bioavailability, bioaccumulation, toxicity behavior, etc. of free metal ions, the evaluation of metal complexes in ecosystems is also a research, technological and financial issue of great importance [9].

The most common way to detect the presence of heavy metals is the use of physicochemical analysis of water or sediment samples [10]. However, due to the complex nature of environmental wastes, a short-term toxicity based bioassays may increase the efficiency of the chemical analytical techniques [10, 11]. Biological systems are important indicators of aquatic pollution in combination with the pre-mentioned characterizations [10]. Therefore, biological assays, such as *Allium cepa* and *Artemia salina* assays, were already used for detecting the genotoxicity [12, 13]. *Allium cepa* assay has been standardized by the United Nations Environment Program and the Environmental Protection Agency’s (EPA) international programs as bio-indicator for the risk assessment of heavy metals ions contamination and the determination of their genotoxicity [14, 15]. *A. cepa* assay enables the detection of different genetic endpoints for the cytotoxic, genotoxic, clastogenic and aneugenic effects of toxic substances [12]. The Mitotic index (MI), chromosomal abnormalities (CA), nuclear abnormalities (NA) frequencies and micronucleus (MN) can be used as indicators to assess the cytotoxicity of several agents [12]. *Artemia salina* is a zooplanktonic crustacean [13] and it can be found in a variety of seawater systems [13]. *A. salina* interacts with the aquatic environment and faces high risk exposure to contaminants [13]. For the toxicological evaluation, endpoints can be used, such as hatching, mortality, swimming, morphology and biomarkers [13]*.* Moreover*,* nauplii of the brine shrimp have been considered a simple and suitable model system for acute toxicity tests [13].

Within this review, the reports on the assessment of the biological effect of metal ions and their complexes using the *Allium cepa* and *Artemia salina* assays are critically discussed. Reports that include metal ions and complexes interaction with either *Allium cepa* or *Artemia salina* bio-indicators are included in the review. Metal ions of the groups 11 (Cu, Ag, Au), 12 (Zn, Cd, Hg), 14 (Sn, Pb) and 15 (Sb, Bi), was selected during the literature search. Therefore, all works published on this subject were included to the best of our knowledge.

## Results and discussion

### *Allium cepa *assay

The need for in vivo sensitive tools for toxicity monitoring is increasing and experimental models, besides animals, are becoming popular. *A. cepa* exhibits many similarities with the mammalian test models [13]. The assay based on this plant is useful for the detection and the evaluation of the effects or the presence of a contaminant, such as metal ions [13]. The influence of such contaminants on the MI and the DNA damage (CA, NA, MN) is estimated after the 24 h or 48 h exposure of *A. cepa* roots in different concentrations of the contaminant [13].

**Fig. 1:**
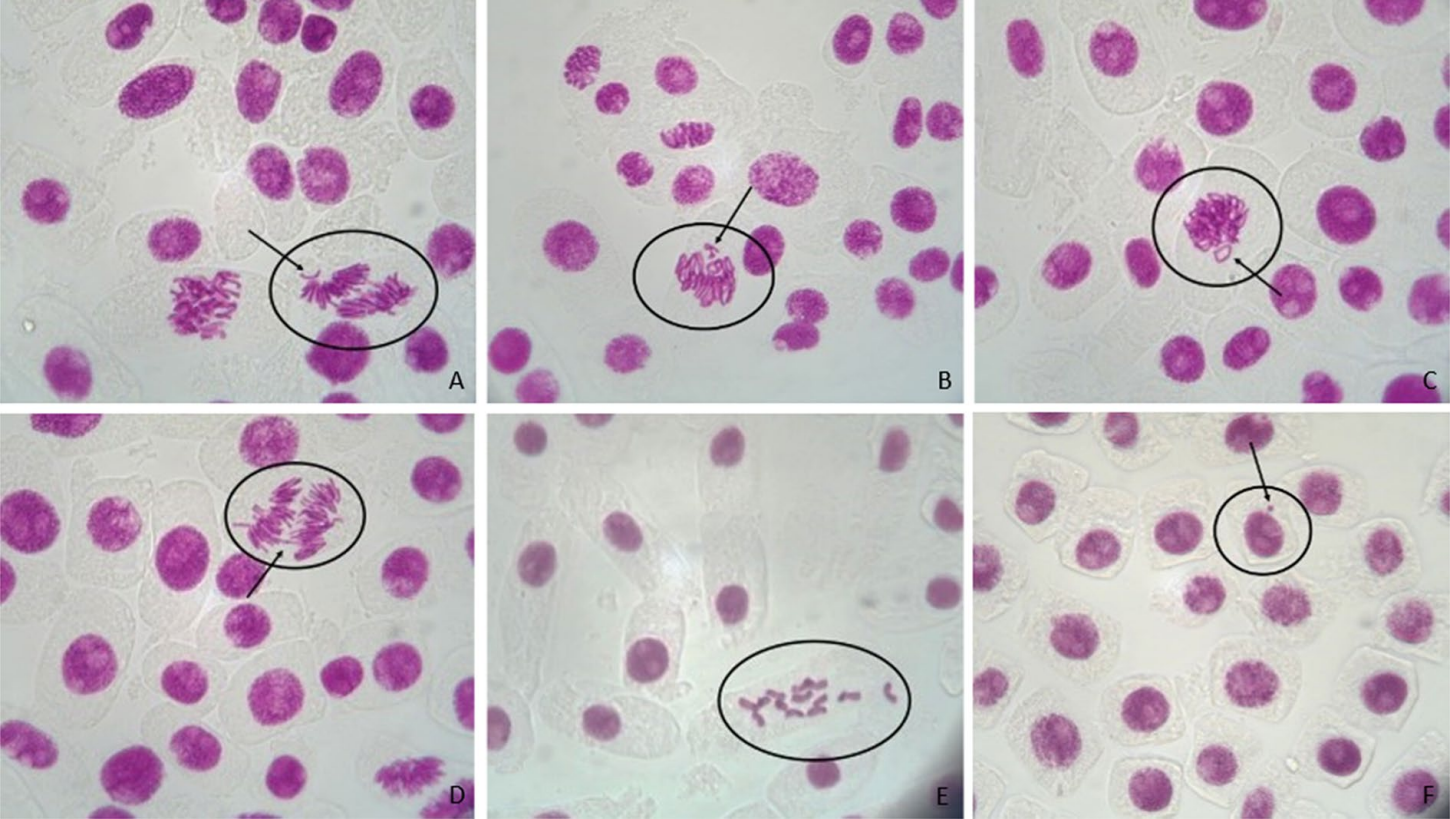
CA observed in *A. cepa* root cells. **A** Chromosomal loss or fragment in anaphase, **B** Chromosomal loss or fragment in metaphase, **C** Chromosomal loss or fragment in prophase, **D** Chromosomal bridge in anaphase, **E** C-mitosis and **F** Micronucleus

This review examines the effects of heavy metal ions on the MI and the CA, which were observed in the onion cells. The MI% is defined as the ratio between the cells in a population undergoing mitosis to the cells not undergoing mitosis [16]. CAs emerge from the exposure to physical or chemical agents and are presented as changes in chromosomal structure or in the total number of chromosomes [17]. MN is arisen from the development of CA, and result from damages, not or wrongly repaired, in the parental cells [14]. More specifically, chromosomal loses and fragments, which are not included in the main nucleus, form a smaller structure, which is called micronucleus [14]. CAs are chromosomal bridges, chromosomal loss, stickiness, c-mitosis, etc. [17]. The first two belongs to clastogenic aberrations, along with chromosomal breaks, while the others are included to physiological aberrations [18]. Stickiness is emerged from the high condensation of chromosomes or the depolymerization of DNA and its outcome is cell death in most cases [18]. C-mitosis is the scattering of the chromosomes all over the cell because of the prevention of the formation of spindle fibers due to colchicines [18]. Vagrant and laggard/lagging chromosomes are also physiological aberrations [18]. The first one describes the movement of a chromosome ahead of its group, leading to unequal separation, while the second refers to the chromosomes that fail to attach to the spindle fiber [18]. Another chromosomal aberration is called clumping and reports the appearance of a cluster of chromosomes in different phases of cell cycle [19]. Chromosomal adherence is another term for approximately the same effect, namely the presence of attached chromosomes [14]. Finally, tripolar mitosis describes the separation of chromosomes in three poles due to the presence of three strands of a division spindle [20]. Some common CA types are presented in Fig. 1.

To compare the MI% values of the *A. cepa* root cells after their exposure to different metal complexes or salts, we introduce a new term the % Mitotic Index Alteration upon their incubation in a particular concentration of the agent (% MIA(C)). This is necessity due to the control samples quality diversity used as well as the variety of *A. cepa* bulb types. Thus, % MIA(C) corresponds to a specific MI % control value at a specific concentration (C).

$$\% MIA\left(C\right)= \frac{{100 \times MI\%(C)}_{sample}}{{MI\%}_{control}}$$

% MIA(C) indicates the percentage of the cells which undergo mitosis in a specific concentration, in respect to the corresponding percentage in the control sample. So, a reduction in % MIA(C) reflects the reduction of the number of cells undergoing mitosis and, consequently, the decrease of cell viability. According to ISO 10993-5:2009, a substance is considered as non-toxic, if it promotes the death of < 30% of the cells (viability ≥ 70%) [21, 22]. We extend here the assumption that if an agent introduces % MIA(C) ≥ 70%, then it is considered as a non-toxic as well. It is pointed out that the samples numbering shows their ingredients, in a particular concentration.

### Group 10 metals (Ni, Pd, Pt) complexes

*Platinum:* Samples of platinum(II) compounds with the thiosemicarbazone 1-(1H-Benzimidazol-2-yl)ethan-1-one thiosemicarbazone (BzimetTSCH), formuale [Pt(BzimetTSC)Cl]·2H_2_O (**1**) and [Pt(BzimetTSC)(TPP)]Cl·H_2_O·MeCN (**2**) (TPP = triphenylphophine) were examined for their in vivo toxicity at 3 (**1.1** and **2.1**), 30 (**1.2** and **2.2**) and 300 (**1.3** and **2.3**) μM (Table 1). The range of % MIA values lies between 54.0 and 73.0% for the samples **1.1–1.3**, while in the case of the samples **2.1–2.3**, is between 73.0 and 64.0% (Table 1). In the case of the samples **2.2–2.3,** the CA are increased in contrast to control [23].

**Table 1:** Metal complexes tested against *Allium cepa*

**Code**	**Molecular formula**	**Molecular weight (g/mol)**	***C* (μM)**	**MI (%)**		**MIA%**		**CA (%)**		**Refs.**
				24 h	48 h	24 h	48 h	24 h	48 h	
**1.1**	[Pt(BzimetTSCH)Cl]·2H_2_O	498.86	3		4.7		70%		0.9	[23]
**1.2**	[Pt(BzimetTSCH)Cl]·2H_2_O	498.86	30		4.9		73%		1.3	[23]
**1.3**	[Pt(BzimetTSCH)Cl]·2H_2_O	498.86	300		3.6		54%		1.8	[23]
**2.1**	[Pt(BzimetTSCH)(tpp)]Cl·H_2_O·MeCN	784.17	3		4.4		66%		1.2	[23]
**2.2**	[Pt(BzimetTSCH)(tpp)]Cl·H_2_O·MeCN	784.17	30		4.9		73%		2.1	[23]
**2.3**	[Pt(BzimetTSCH)(tpp)]Cl·H_2_O·MeCN	784.17	300		4.3		64%		2.7	[23]
**2.4**	ddH_2_O				6.7				1.3	[23]
**3.1**	3-H2TPtPyP	2165.58	0.6		57.0*		104%			[24]
**3.2**	3-H2TPtPyP		1.1		54.0.		98%			[24]
**3.3**	**3-H2TPtPyP**		2.2		52		95%			[24]
**3.4**	**3-H2TPtPyP**		5.5		56		102%			[24]
**3.5**	**Control**				55					[24]
**4.1**	[Pt(NH_3_)_2_Cl_2_]	300.05	0.1	11		81%				[25]
**4.2**	[Pt(NH_3_)_2_Cl_2_]	300.05	0.5	10.5		78%				[25]
**4.3**	[Pt(NH_3_)_2_Cl_2_]	300.05	1	2.5		19%				[25]
**4.4**	[Pt(NH_3_)_2_Cl_2_]	300.05	5	1.4		10%				[25]
**4.5**	Control cisplatin			13.5						[25]
**5.1**	Carboplatin	371.25	0.5	19		136%				[25]
**5.2**	Carboplatin	371.25	1	15		107%				[25]
**5.3**	Carboplatin	371.25	10	12.5		89%				[25]
**5.4**	Carboplatin	371.25	50	14.5		104%				[25]
**5.5**	Carboplatin	371.25	100	13		93%				[25]
**5.6**	Control carboplatin			14						[25]
**6.1**	Silica-NMP-Cu		1.0 *^2^	0.9		96.8		0.05		[26]
**6.2**	Silica-NMP-Cu		3.0 *^2^	0.9		93.6		0.06		[26]
**6.3**	Silica-NMP-Cu		6.0 *^2^	0.9		90.4		0.06		[26]
**6.4**	ddH_2_O									[26]
**7.1**	([Ag_3_(Gly)_2_NO_3_]_n_)	542.75	24		8.2		85%		0.5	[27]
**7.2**	([Ag_3_(Gly)_2_NO_3_]_n_)	542.75	49		8.9		92%		0.3	[27]
**7.3**	([Ag_3_(Gly)_2_NO_3_]_n_)	542.75	98		6.6		68%		0.4	[27]
**7.4**	ddH_2_O				9.7					[27]
**8.1**	{[Ag(CIPH)_2_]NO_3_·0.75MeOH·1.2H_2_O	872.78	0.3		6.6		99%		1	[16]
**8.2**	{[Ag(CIPH)_2_]NO_3_·0.75MeOH·1.2H_2_O	872.78	3		6.2		93%		0.6	[16]
**8.3**	{[Ag(CIPH)_2_]NO_3_·0.75MeOH·1.2H_2_O	872.78	30		6		90%		0	[16]
**8.4**	ddH_2_O				6.7				0.5	[16]
**9.1**	{[Ag_6_(*μ*_3_-Hmna)_4_(*μ*_3_-mna)_2_]^2−^·[(Et_3_NH)+]_2_·(DMSO)_2_·(H_2_O)}	1948.7	3		4.1		82%		0.5	[28]
**9.2**	{[Ag_6_(*μ*_3_-Hmna)_4_(*μ*_3_- mna)_2_]^2−^·[(Et_3_NH)+]_2_·(DMSO)_2_·(H_2_O)}	1948.7	30		4.5		90%		0.4	[28]
**9.3**	{[Ag_6_(*μ*_3_-Hmna)_4_(*μ*_3_- mna)_2_]^2−^·[(Et_3_NH)+]_2_·(DMSO)_2_·(H_2_O)}	1948.7	300		4.7		94%		0.8	[28]
**9.4**	ddH_2_O				5				0.4	[28]
**10.1**	[Ag(salH)]_2_	489.96	3		7.2		107%		0.6	[29]
**10.2**	[Ag(salH)]_2_	489.96	30		6.7		100%		0.3	[29]
**10.3**	[Ag(salH)]_2_	489.96	300		2.3		34%		0.8	[29]
**10.4**	ddH_2_O	-	-		6.7		100%		0.4	[29]
**11.1**	[AgBr(*μ*_2_-S-MMI)(TPP))]_2_	1128.44	3		6.8		117%		1.4	[30]
**11.2**	[AgBr(*μ*_2_-S-MMI)(TPP))]_2_	1128.44	30		5.3		91%		0.3	[30]
**11.3**	[AgBr(*μ*_2_-S-MMI)(TPP))]_2_	1128.44	300		5.3		91%		0.7	[30]
**12.1**	[AgCl(TPP)_2_(MMI)]	782.04	3		4.8		83%		1.1	[30]
**12.2**	[AgCl(TPP)_2_(MMI)]	782.04	30		5.2		90%		0.6	[30]
**12.3**	[AgCl(TPP)_2_(MMI)]	782.04	300		5.5		95%		1	[30]
**12.4**	ddH_2_O				5.8				1.1	[30]
**13.1**	[Ag(SCP)]	391.58	3.2	39.6		71%				[31]
**13.2**	[Ag(SCP)]	391.58	16	38		68%				[31]
**13.3**	[Ag(SCP)]	391.58	63.8	28.4		51%				[31]
**13.4**	[Ag(SCP)]	391.58	159.6	26		47%				[31]
**13.5**	[Ag(SCP)]	391.58	319.2	23.6		42%				[31]
**13.6**	Control - [Ag(SCP)]	–	–	55.6		100%				[31]
**14.1**	(Ag_3_[Ag(SCN)_3_(SCP)]·H_2_O)	907.41	1.4	37		101%				[31]
**14.2**	(Ag_3_[Ag(SCN)_3_(SCP)]·H_2_O)	907.41	6.9	47		129%				[31]
**14.3**	(Ag_3_[Ag(SCN)_3_(SCP)]·H_2_O)	907.41	27.6	35.9		98%				[31]
**14.4**	(Ag_3_[Ag(SCN)_3_(SCP)]·H_2_O)	907.41	68.9	27.8		76%				[31]
**14.5**	(Ag_3_[Ag(SCN)_3_(SCP)]·H_2_O)	907.41	137.8	12		33%				[31]
**14.6**	Control - [(Ag_3_[Ag(SCN)_3_(SCP)]·H_2_O)	–	–	36.6		100%				[31]
**15.1**	Ag(SDM)	417.19	31	–		176%		–		[32]
**15.2**	Ag(SDM)	417.19	153			99%		–		[32]
**15.3**	Ag(SDM)	417.19	306	–		100%		–		[32]
**15.4**	Ag(SDM)	417.19	458			81%		–		[32]
**15.5**	Ag(SDM)	417.19	611			103%		–		[32]
**16.1**	Ag3SDM(SCN)2]·H2O	767.12	16.4			131%		–		[32]
**16.2**	Ag_3_SDM(SCN)_2_]·H_2_O	767.12	82			118%		–		[32]
**16.3**	Ag_3_SDM(SCN)_2_]·H_2_O	767.12	164			126%		–		[32]
**16.4**	Ag_3_SDM(SCN)_2_]·H_2_O	767.12	246			74%		–		[32]
**16.5**	Ag_3_SDM(SCN)_2_]·H_2_O	767.12	328			120%		–		[32]
**16.6**	Control for [Ag(SDM)],Ag_3_SDM(SCN)_2_]·H_2_O	–	–					–		[32]
**17.1**	Ag_2_(SDM)_2_*o*-phen] ·H_2_O	1032.61	12.5			97%		–		[32]
**17.2**	Ag_2_(SDM)_2_*o*-phen] ·H_2_O	1032.61	62.6			0%		–		[32]
**17.3**	Ag_2_(SDM)_2_*o*-phen] ·H_2_O	1032.61	125			0%		–		[32]
**17.4**	Ag_2_(SDM)_2_*o*-phen] ·H_2_O	1032.61	188			0%		–		[32]
**17.5**	Ag_2_(SDM)_2_*o*-phen] ·H_2_O	1032.61	250			0%		–		[32]
**17.6**	Control for Ag_2_(SDM)_2_*o*-phen] ·H_2_O	–	–					–		[32]
**18.1**	[Ag_2_(SMX)_2_]·H_2_O	766.34	1.6	37.8		78%				[33]
**18.2**	[Ag_2_(SMX)_2_]·H_2_O	766.34	8.2	37.7		77%				[33]
**18.3**	[Ag_2_(SMX)_2_]·H_2_O	766.34	32.6	36.2		74%				[33]
**18.4**	[Ag_2_(SMX)_2_]·H_2_O	766.34	81.2	28.4		58%				[33]
**18.5**	[Ag_2_(SMX)_2_]·H_2_O	766.34	326.2	17.2		35%				[33]
**18.6**	Control [Ag_2_(SMX)_2_]·H_2_O			48.7						[33]
**19.1**	[Ag_4_(SCN)_3_(SMX)]·H_2_O	890.03	1.3	45.6		98%				[33]
**19.2**	[Ag_4_(SCN)_3_(SMX)]·H_2_O	890.03	6.4	51.1		110%				[33]
**19.3**	[Ag_4_(SCN)_3_(SMX)]·H_2_O	890.03	25.5	37.3		67%				[33]
**19.4**	[Ag_4_(SCN)_3_(SMX)]·H_2_O	890.03	63.8	31		41%				[33]
**19.5**	[Ag_4_(SCN)_3_(SMX)]·H_2_O	890.03	280.9	19		41%				[33]
**19.6**	Control [Ag_4_(SCN)_3_(SMX)]·H_2_O			46.4						[33]
**20.1**	[Au(tpp)Cl]	494.7	3	6.7		102%		0.3		[34]
**20.2**	[Au(tpp)Cl]	494.7	30	3.7		56%		0.3		[34]
**20.3**	[Au(tpp)Cl]	494.7	300	3.7		56%		1.5		[34]
**20.4**	ddH_2_O			6.6		22%		0.6		[34]
**21.1**	ZnO-NPs		5*^3^	60		42%				[35]
**21.2**	ZnO-NPs		50 *^3^	31		22%				[35]
**21.3**	control			144						[35]
**22.1**	Zn(NO_3_)_2_	189.36	0.77	110		183%		0		[36]
**22.2**	Zn(NO_3_)_2_	189.36	7.7	41		68%		2		[36]
**22.3**	Zn(NO_3_)_2_	189.36	76.9	20		33%		2.3		[36]
**23.1**	Cd(NO_3_)_2_	236.42	0.44	53		88%		0.8		[36]
**23.2**	Cd(NO_3_)_2_	236.42	4.4	32		53%		1.6		[36]
**23.3**	Cd(NO_3_)_2_	236.42	44.4	16		27%		1.9		[36]
**23.4**	control			62						[36]
**24.1**	CdCl_2_	183.31	50	39.2	41.7	74%	91%	5.9	2.9	[37]
**24.2**	CdCl_2_	183.31	80	49.9	49.8	94%	109%	4.7	4.5	[37]
**24.3**	CdCl_2_	183.31	100	35.7	44.2	67%	96%	20	2	[37]
**24.4**	dH_2_O		-	53.2	45.9	100%	100%	0	0	[37]
**25.1**	PH_3_Sn(CA)	757.5	0.1		2.7		77%		0.6	[38]
**25.2**	PH_3_Sn(CA)	757.5	1		3.5		100%		2.8	[38]
**25.3**	PH_3_Sn(CA)	757.5	10		1.3		37%		2.5	[38]
**26.1**	n-BuSn(CA)	697.5	0.1		4		114%		0.7	[38]
**26.2**	n-BuSn(CA)	697.5	1		3.5		100%		1.7	[38]
**26.3**	n-BuSn(CA)	697.5	10		2.2		63%		1.2	[38]
**27.1**	Ph_2_Sn(CA)_2_	1087.9	0.1		2.8		80%		0.3	[38]
**27.2**	Ph_2_Sn(CA)_2_	1087.9	1		2.7		77%		0.7	[38]
**27.3**	Ph_2_Sn(CA)_2_	1087.9	10		2.6		74%		1	[38]
**28.1**	(n-Bu)_2_Sn(CA)_2_	1047.9	0.1		3.6		103%		1	[38]
**28.2**	(n-Bu)_2_Sn(CA)_2_	1047.9	1		3.7		106%		1.4	[38]
**28.3**	(n-Bu)_2_Sn(CA)_2_	1047.9	10		3.5		100%		1.3	[38]
**28.4**	ddH_2_O				3.5		100%		0.6	[38]
**29.1**	Pb(NO_3_)_2_	331	0.24	50		83%		1.1		[36]
**29.2**	Pb(NO_3_)_2_	331	2.41	22		36%		2.6		[36]
**29.3**	Pb(NO_3_)_2_	331	24.1	10		17%		3.3		[36]
**29.4**	control			60						[36]
**30.1**	{[SbBr(Me_2_DTC)_2_]_n_}	441.11	0.01		10.5		135%		0.7	[17]
**30.2**	{[SbBr(Me_2_DTC)_2_]_n_}	441.11	0.1		10.8		138%		0.5	[17]
**30.3**	{[SbBr(Me_2_DTC)_2_]_n_}	441.11	1		8.4		108%		0.5	[17]
**31.1**	{[SbI(Me_2_DTC)_2_]_n_}	499.11	0.01		2.6		33%		0.8	[17]
**31.2**	{[SbI(Me_2_DTC)_2_]_n_}	499.11	0.1		6.4		82%		0.5	[17]
**31.3**	{[SbI(Me_2_DTC)_2_]_n_}	499.11	1		5.1		65%		1.9	[17]
**32.1**	{[(Me2DTC)_2_Sb(μ_2_-I)Sb(Me_2_DTC)_2_]	1232	0.01		4.4		56%		1.8	[17]
**32.2**	{[(Me2DTC)_2_Sb(μ_2_-I)Sb(Me_2_DTC)_2_]	1232	0.1		8.3		106%		1.2	[17]
**32.3**	{[(Me2DTC)_2_Sb(μ_2_-I)Sb(Me_2_DTC)_2_]	1232	1		1.6		21%		1.7	[17]
**32.4**	Control				7.8		100%		0.5	[17]

^*^Exposed for 96 h days, *^2^ mg/L, *^3^ μg/mL

The in vivo* toxicity* of tetrapyridylporphyrin containing four chloro(2,2′-bipyridine)platinum(II) complex (3-H_2_TPtPyP) (**3.1–3.4**) attached at the meta position of the peripheral pyridine ligand was tested at 0.6–5.5 μΜ (Table 1). The sample shows no in vivo toxicity since the %MIA is almost 100 at the highest concentration (**3.4**), which is in consistent with the % root length [24].

*A. cepa* bulbs were exposed for 24 h to aqueous solutions of cisplatin (**4.1–4.4**) and carboplatin (**5.1–5.5**) (Table 1). The % MIA values showed that cisplatin was toxic at the concentration of 1 and 5 μM, whereas carboplatin was not toxic in the tested concentrations [25].

### Group 11 metals (Cu, Ag, Au) complexes

Copper*: A. cepa* bulbs were incubated with samples of nano-silica Schiff-base Cu(II) (Silica-NMP-Cu, NMP = N-methyl pyrrolidone) (1.50 (**6.1**), 3.00 (**6.2**) and 6.00 (**6.3**) mg/L) (Table 1). The samples numbering corresponds to their ingredients, in a particular concentration. For example, the code **6.1** refers to the sample of Silica-NMP-Cu at the concentration of 1.50 mg/L. The % MIA of the Cu(II) was in the range of 90.4–96.8%, suggesting that its in vivo genotoxicity is low (Table 1). The percentage of CAs was similarly to those of control ones [26].

Silver*: A. cepa* bulbs were incubated with [Ag_3_(Gly)_2_NO_3_]_n_ (GlyH = glycine) (AGGLY) at the concentrations range of 24–98 μM (**7.1–7.3**) (Table 1) [23]. The % MIA values varied from 68 (**7.3**) to 92 (**7.2**) %. The CA was 0.5% for **7.1**, 0.33% for **7.2** and 0.41% for **7.3**. These values suggest a low in vivo toxic activity (ISO 10993–5:2009) of [Ag_3_(Gly)_2_NO_3_]_n_ [27].

The combination of the antibiotic ciprofloxacin (CIPH) with silver(I) ions resulted to the {[Ag(CIPH)_2_]NO_3_•0.75MeOH•1.2H_2_O (CIPAG) [16]. The silver(I) compound was assessed for its in vivo toxicity through *A. cepa* test in different concentrations (0.3 (**8.1**), 3 (**8.2**) and 30 (**8.3**) μM). The %MIA values were 90 (**8.3)**–99% (**8.1**) (Table 1). The CA values were 0.0–1.0% (**8.3–8.1**) (Table 1). Thus, neither % MIA nor CA are affected by the presence of the silver compound [16].

The in vivo toxicity of the silver(I) compound of formula {[Ag_6_(*μ*_3_-Hmna)_4_(*μ*_3_- mna)_2_]^2−^·[(Et_3_NH) +]_2_·(DMSO)_2_·(H_2_O)} (H_2_mna = 2-mercapto-nicotinic acid) (AGMNA) was tested in the concentrations of 3 (**9.1**), 30 (**9.2**) and 300 (**9.3**) μM (Table 1) [28]. The cell division rate of *A. cepa* root cells was not affected by the presence of AGMNA since the range of % MIA lies between 82 and 94%. The same trend was followed by CAs, (0.4% (**9.2**) to 0.8% (**9.3**)). Therefore, AGMNA has no in vivo toxic or mutagenic effects according to ISO 10993-5:2009 [21, 22].

The in vivo toxicity of [Ag(salH)]_2_ (salH_2_ = salicylic acid) (AGSAL) (3 (**10.1**), 30 (**10.2**) and 300 (**10.3**) μM) is tested by *A. cepa* assay (Table 1) [29]. No variation in % MIA values was observed at the concentrations up to 30 μM (Table 1). However, when *Allium cepa* were incubated with AGSAL at the concentration of 300 μM, the % MIA values reduced to the 34%, while the CAs doubled in respect to those observed in lower concentrations. Chromosome adherences or chromosome losses were the most common types of CAs [29].

Samples of two silver(I) compounds [AgBr(*μ*_2_-S-MMI)(TPP))]_2_ (**11.1–11.3**) and [AgCl(TPP)_2_(MMI)] (**12.1–12.3**) (TPP = triphenylphosphine, MMI = 2-mercapto-1-methyl-imidazole or methimazole) were evaluated through *A. cepa* assay (Table 1) [30]. No effect in % MIA was observed upon their incubation with **11.1–11.3** and **12.1–12.3**. The absence of variations in the CA values indicates the absence of in vivo toxic behavior [30].

The samples of the silver(I) compounds [Ag(SCP)] (**13.1–13.5**) and (Ag_3_[Ag(SCN)_3_(SCP)]·H_2_O) (SCP = Sulfachloropyridazine) (**14.1–14.5**) were tested with *A. cepa* assay (Table 1). In vivo toxicity was detected considering both % MIA and root lengths, after their exposure to silver complexes solutions for 24 h (Table 1) [31]. Thus, the % MIA in the case of **13.2–13.5** lies between 42 and 68%. This is consistent with the high percentage reduction of the root length (20–60%), toward the corresponding of the control sample. However, the presence of SCN^−^ anion in the coordination sphere increases the in vivo toxic limit at the concentration of 1.4 mM (**14.5**), with the % MIA value to be 33% for this concentration [31].

Similarly, the samples of compounds Ag(SDM) (**15.1–15.5**), Ag_3_SDM(SCN)_2_]·H_2_O (**16.1–16.5**) and Ag_2_(SDM)_2_*o*-phen] ·H_2_O (**17.1–17.5**) (SDM = sulfadimetoxine, phen = 1,10-phenathroline) have also been evaluated in the same manner. The % MIA values suggest no in vivo toxic behavior in the case of **15.1–15.5** and **16.1–16.5** (Table 1) [32]. However, by taken into consideration the % root length variations, an in vivo toxicity might be proposed for these samples, but the confidence limits of these values exceed or lie to the values themselves (Table 1) [32]. The null % MIA values in the case of samples **17.2–17.5** show in vivo toxicity since there is no cell division [32].

The in vivo toxicity of the samples of Ag(I) complexes with sulfamoxole (SMX), formulae [Ag_2_(SMX)_2_]·H_2_O (**18.1–18.5**) and [Ag_4_(SCN)_3_(SMX)]·H_2_O (**19.1–19.5**) was also examined (Table 1). The % MIA values of 58% and 67% suggest that these complexes were toxic at concentrations higher than 81.2 and 25.5 μΜ, respectively. In addition, the root length was affected at concentrations higher than 32.6 and 6.4 μΜ, respectively [33].

Gold: The genotoxicity of gold complex [Au(TPP)Cl] (TPP = triphenylphosphine) (**20.1–20.3**) was tested via *A. cepa* root cells, in three different concentrations (3 (**20.1**), 30 (**20.2**) and 300 (**20.3**) μM) (Table 1) [34]. The % MIA values of **20.2** and **20.3** were 56% indicating in vivo toxicity, which is also concluded by high % CA values (Table 1) [34].

### Group 12 metals (Zn, Cd, Hg) Complexes

Zinc*:* The effects of 5 μg/mL and 50 μg/mL ZnO-NPs (**21.1–21.2**) on root growth of *A. cepa* were investigated after 36 h incubation (0 h, 12 h, 24 h and 36 h) (Table 1). The root length significantly decreased at both concentrations. Concerning the effect of the exposure time, the root length slightly increased from 0 to 36 h at 5 μg/mL ZnO NPs, while no growth observed after 0 h to 36 h incubation with 50 μg/mL ZnO NPs. The corresponding % MIA values revealed that these concentrations were toxic after 12-h, 24-h and 36-h incubation [35].

The incubation of *A. cepa* bulbs in zinc (in the form of zinc nitrate) at 0.77–76.92 μΜ (**22.1–22.3**) resulted in the variation of % MIA (183%, 68% and 33%) (Table 1). Thus, the in vivo toxicity of Zn ions appeared in concentrations higher than 7.7 μΜ. The CAs are increased in the same concentrations (0%, 2% and 2.3%) accordingly [36].

Cadmium*: A. cepa* bulbs were incubated in 0.44, 4.45 and 44.48 μΜ cadmium (in the form of cadmium nitrate) (**23.1–23.3** respectively) and the % MIA values were 88%, 53% and 27%, respectively (Table 1). Taking into account that if % MIA is lower than 70%, the metal ions are deemed toxic, the in vivo toxicity of Cd ions in concentrations higher than 0.21 μΜ is concluded. The CAs were 0.8%, 1.6% and 1.9%, respectively, leading to the same conclusion [36].

*A. cepa* cells were used to evaluate the in vivo genotoxicity of CdCl_2_ in different concentrations 50 (**24.1**), 80 (**24.2**) and 100 (**24.3**) μΜ upon their exposure for (2, 24 and 48 h) (Table 1). No in vivo toxicity was detected from these samples toward *A. cepa* cells at incubation periods (24 and 48 h) (ISO 10993-5:2009 [21]) [37]. However, an increasing in the % CA was observed in the case of **24.3**. The most common CAs that were observed were chromosomal bridges, breaks, stickiness and clumping [37]. Given that cadmium(II) are among the heavy metals that causes genotoxicity, mutagenicity, and carcinogenicity in humans and other living organisms, the low or no toxicity which is observed for the **24.1–24.2**, should not only be attributed to the low concentration but to the type of bulb used, as well [37].

### Group 14 metals (Sn, Pb) complexes

Organotins*:* Organotin compounds derived from cholic acid (CAH) R_3_Sn(CA) [R = Ph- (**25**), *n*-Bu- (**26**)] and R_2_Sn(CA)_2_ [R = Ph- (**27**) and *n*-Bu- (**28**)] were evaluated for their in vivo toxicity at the concentrations 0.1 μM (**25.1, 26.1**, **27.1, 28.1**), 1 μM (**25.2, 26.2**, **27.2, 28.2**) and 10 μM (**25.3, 26.3**, **27.3, 28.3**) (Table 1). The diorganotin compounds show no in vivo genotoxicity in contrast to tri-organotin ones. The % MIA in the case of diorganotin is in the range of 74–106% while those of tri-organotin in between 37 and 114% [38].

Lead*:* The % MIA values of *A. cepa* cells upon their treatment with 0.24, 2.41 and 24.13 μΜ Pb ions (in the form of Pb(NO_3_)_2_) (samples id: **29.1–29.3** respectively) were 82%, 36% and 16% (Table 1). Based on this, the in vivo toxicity of Pb is concluded over 2.41 μΜ. The corresponding CAs were 1.1%, 2.6% and 3.3% [36].

### Group 15 metals (Sb, Bi) complexes

Antimony*:* Three antimony compounds with the formulae {[SbBr(Me_2_DTC)_2_]_n_} (**30**), {[SbI(Me_2_DTC)_2_]_n_} (**31**) and {[(Me_2_DTC)_2_Sb(*μ*_2_-I)Sb(Me_2_DTC)_2_] (**32**) (Me_2_DTC = dimethyldithiocarbomate) were evaluated for their in vivo toxicity. Samples at concentrations 0.01 (**30.1, 31.1, 32.1**), 0.10 (**30.2, 31.2, 32.2**) and 1.00 (**30.3, 31.3, 32.3**) μM were used (Table 1). The compound of antimony bromide exhibits no genotoxicity (% MIA 108–135% **30.1–30.3**) in contrast to antimony iodides (% MIA 33–82% (**31.1–31.3**) and 21–106% (**32.1–32.3**) respectively). Consequently, the % CA in the case of samples **31.1–31.3** and **32.1–32.3** is increased. Sticky, bridges and vagrant chromosomes were commonly observed on the samples [17].

### *Artemia salina *assay

**Fig. 2:**
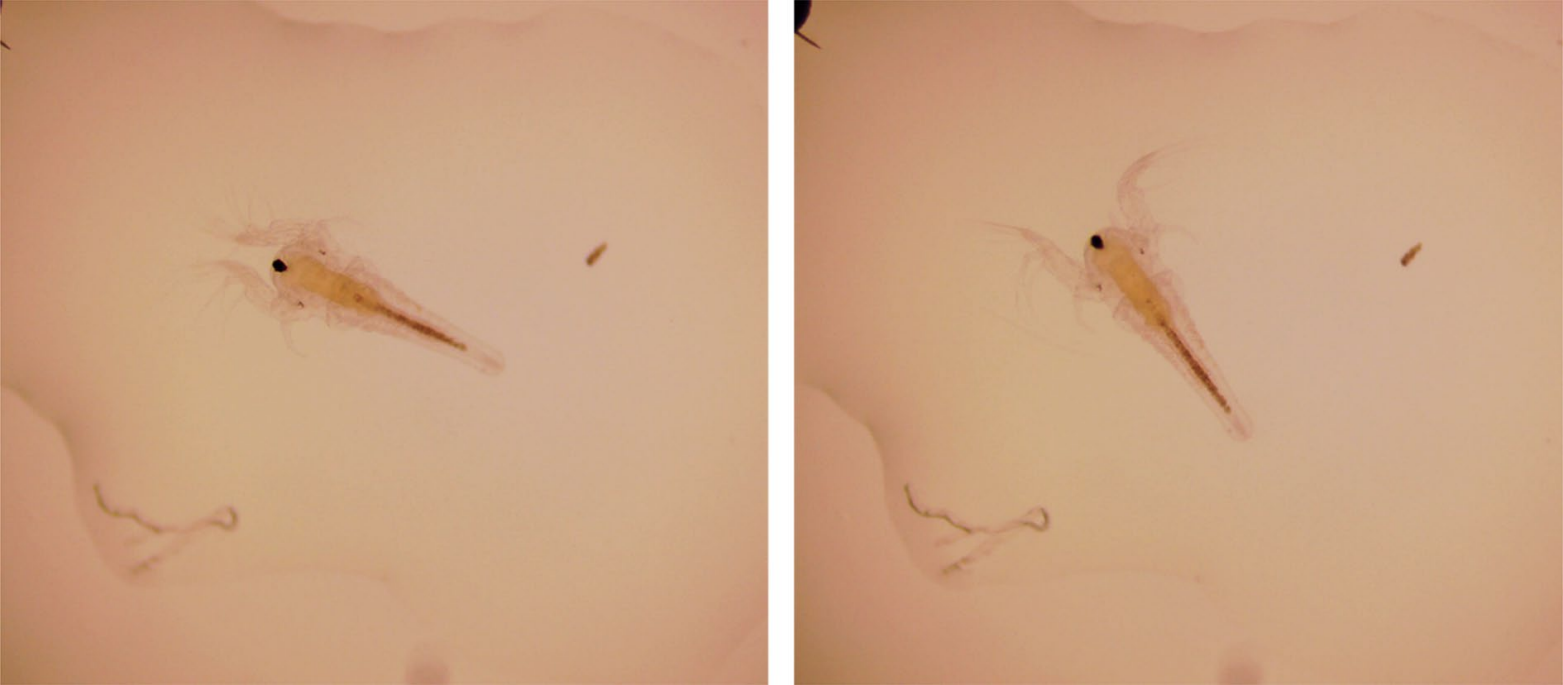
Nauplii brine shrimp of *Artemia salina*

Along with *A. cepa*, *Artemia salina* is also a biological model widely used for acute toxicity tests [13] (Fig. 2). The nauplii of the zooplanktonic crustacean is highly sensitive to contaminants in the aquatic environment [13]. The advantages of the usage of *A. salina* in genotoxicity tests are its short lifetime, its availability, low cost and easy and safe use and its high offspring number [13]. The examined indicators in this assay are the Lethal Concentration (LC_50_ in mM) or Dose (LD_50_ in mg/mL) that eliminates the 50% of the nauplii. *A salina* is considered as dead when it exhibits no any internal or external movement for 10 s of observation [13].

### Group 10 metals (Ni, Pd, Pt) complexes

Nickel*:* The LD_50_ value of nickel metal organic framework (Ni-MOFs) (**33**) was estimated 138.33 μg/mL (Table 2) [39].

**Table 2:** LC_50_ and LD_50_ values of metal complexes and metal salts tested with *Artemia salina* assay

Code	Molecular formula	Molecular weight (g/mol)	LC_50_ (mM)	LD_50_ (mg/mL)	Refs.
33	Ni-MOFs	-	-	0.138	[39]
34	[NiL(Cl)_2_]	459.9	0.255	0.117	[34]
35	[Ni_2_L^1^_2_(*μ*-1,1-N_3_)_2_(N_3_)_2_]·4H_2_O	838.12	0.860	0.720	[41]
36	([Ni_2_L^2^_2_(*μ*-1,1-N_3_)_2_(N_3_)_2_]·6H_2_O)	862.09	0.820	0.710	[41]
37	[Ni(L^1^)_2_Cl_2_]	842.49	> 1.19	> 1.00	[42]
38	[Ni(L^2^)_2_Cl_2_]	630.14	> 1.59	> 1.00	[42]
39	[Ni(L^3^)_2_Cl_2_]	686.25	> 1.46	> 1.00	[42]
40	[Ni(L^4^)_2_Cl_2_]	792.29	> 1.26	> 1.00	[42]
41	[Ni(L^5^)_2_Cl_2_]	658.20	> 1.52	> 1.00	[42]
42	[Ni(L^6^)_2_Cl_2_]	714.30	0.193	0.140	[42]
43	[Ni(H_3_L^1^)(H_2_L^1^)](ClO_4_)_2_·2H_2_O	615.58	0.143	0.088	[43]
44	[Ni(H_3_L^2^)(H_2_L^2^)] (ClO_4_)_2_·H_2_O	625.22	0.154	0.096	[43]
45	[Ni(H_3_L^3^)(H_2_L^3^)](ClO_4_)_2_	731.75	0.087	0.064	[43]
46	[Ni(H_3_L^4^)(H_2_L^4^)](ClO_4_)_2_·2H_2_O	653.67	0.091	0.059	[43]
47	[Ni_2_(HL^1^)_2_]	535.81	0.172	0.092	[43]
48	[Ni_2_(HL^2^)_2_]·H_2_O	557.85	0.157	0.088	[43]
49	[Ni_2_(HL^3^)_2_]	539.84	0.134	0.072	[43]
50	[Ni_2_(HL^4^)_2_]	591.91	0.099	0.059	[43]
51	Ni(BF_4_)_2_·6H_2_O	340.39	0.640	0.220	[41]
52	{[AdNH_3_^+^]·[CuCl_3_]^−^}	322.16	0.428	0.138	[44]
53	[Cu(L^1^)_2_Cl_2_]	847.25	> 1.19	> 1.01	[41]
54	[Cu(L^2^)_2_Cl_2_]	635.00	0.185	0.120	[41]
55	[Cu(L^3^)_2_Cl_2_]	691.10	0.182	0.130	[41]
56	[Cu(L^4^)_2_Cl_2_]	797.14	> 1.250	> 1.00	[41]
57	[Cu(L^5^)_2_Cl_2_]	663.05	> 1.510	> 1.00	[41]
58	[Cu(L^6^)_2_Cl_2_]	719.16	> 1.390	> 1.00	[41]
59	Cu(L^1^-H)_2_(H_2_O)_2_	962.56	> 1039	> 1000	[45]
60	Cu(L^2^-H)_2_(H_2_O)_2_	906.46	601	545	[45]
61	Cu(L^3^-H)_2_(H_2_O)_2_	940.51	484	455	[45]
62	Cu(L^4^-H)_2_(H_2_O)_2_	912.46	> 1096	> 1000	[45]
63	Cu(L^5^-H)_2_(H_2_O)_2_	916.54	606	555	[45]
64	Cu(L^6^-H)_2_(H_2_O)_2_	834.39	627	523	[45]
65	[Cu(L^i^–H)_2_(H_2_O)_2_]	750.31	536	402	[46]
66	[Cu(L^ii^-H)_2_(H_2_O)_2_]	778.36	676	526	[46]
67	Cu(L^iii^-H)_2_(H_2_O)_2_]	806.42	490	395	[46]
68	[Cu(L^1^)_2_Cl_2_]	562.4	> 1.78	> 1.00	[47]
69	[Cu(L^2^)_2_Cl_2_]	590.4	> 1.70	> 1.00	[47]
70	[Cu(L^3^)_2_Cl_2_]	652.4	> 31.53	> 1.00	[47]
71	[Cu(L^4^)_2_Cl_2_]	716.5	0.600	0.430	[47]
72	[Cu(L^5^)_2_Cl_2_]	624.5	0.570	0.354	[47]
73	[Cu(L^6^)_2_Cl_2_]	594.5	> 1.68	> 1.00	[47]
77	Cu(H2Am4DH)Cl_2_]	329.70	0.012	0.004	[49]
78	[Cu(H2Am4Me)Cl_2_]	344.70	0.001	0.0004	[49]
79	[Cu(H2Am4Et)Cl_2_]	357.75	0.002	0.0006	[49]
80	[Cu(2Am4Ph)Cl]	369.3	0.007	0.0027	[49]
81	[CuLCl](NO_3_)	395.27	1.540	0.601	[41]
82	[CuLCl](ClO_4_)	432.71	1.040	0.450	[41]
83	[Cu_2_L^2^(μ-1,1-N_3_)_2_](ClO_4_)_2_)	878.55	0.460	0.404	[41]
84	[CuCl_2_(INH)_2_]·H_2_O	408.71	0.042	0.017	[50]
85	[Cu(NCS)_2_(INH)_2_]·5H_2_O	544.00	0.014	0.008	[50]
86	[Cu(NCO)_2_(INH)_2_]·4H_2_O	493.86	0.494	0.244	[50]
87	[Cu(L^1^)(H_2_O)Cl]	406.40	1.021	0.410	[51]
88	[Cu(L^2^)(H_2_O)Cl]	422.04	2.396	1.010	[51]
89	[Cu(L^3^)(H_2_O)Cl]	386.54	> 2.467	0.950	[51]
90	[Cu(L^4^)(H_2_O)Cl]	414.54	0.748	0.310	[51]
91	[Cu(L^5^)(H_2_O)Cl]	379.34	> 2.515	0.950	[51]
92	[Cu(L^6^)(H_2_O)Cl]	395.04	1.028	0.410	[51]
93	[Cu(L^7^)(H_2_O)Cl]	358.09	> 2.792	1.000	[51]
94	[Cu(L^8^)(H_2_O)Cl]	374.04	> 2.674	1.000	[51]
95	[Cu(L^9^)(H_2_O)Cl]	338.54	0.994	0.340	[51]
96	[Cu(L^10^)(H_2_O)Cl]	366.54	0.873	0.320	[51]
97	[Cu(L^11^)(H_2_O)Cl]	331.04	> 2.891	0.960	[51]
98	[Cu(L^12^)(H_2_O)Cl]	347.04	1.124	0.390	[51]
99	Cu(NO_3_)_2_·3H_2_O	241.6	0.240	0.060	[41]
100	CuCl_2_·2H_2_O	170.48	0.007	0.001	[49]
101	Cu(ClO_4_)_2_·6H_2_O	370.54	0.280	0.104	[41]
102	([Ag(pen)(CH_3_OH)]_2_)	946.49	532	0.504	[52]
103	AgNPs(ORLE)	-	-	217.8	[53]
104	[Zn(valp)_2_phen(H_2_O]	551.0	0.142	0.078	[54]
105	Zn(valp)_2_(bipy)	508.98	0.804	0.409	[54]
106	[Zn(INH)_2_](ClO4)_2_·6H_2_O	646.68	268	0.174	[55]
107	[ZnL^1^(NCS)_2_]·2H_2_O	457.85	1.27	0.581	[41]
108	[ZnL^2^(NCS)_2_]·0.5MeOH	431.83	0.980	0.420	[41]
109	Zn(BF_4_)_2_·6H_2_O	347.08	0.880	0.310	[41]
110	Zn(OAc)_2_·2H_2_O	587.47	1.180	0.690	[41]
111	[CdCl_2_(2,3BTSTCH2)]	505.65	0.300	0.115	[56]
112	[CdBr_2_(2,3BTSTCH2)]	594.65	0.240	0.240	[56]
113	CdHL^3^(NCS)_3_	515.92	0.530	0.273	[41]
114	[CdCl_2_(aphaOEt)(DMF)]	955.33	3.300	3.150	[57]
115	[CdCl_2_(dapha(OEt)_2_)]·1.5H_2_O	1147.49	1.390	1.600	[57]
116	CdCl_2_	183.31	3.030	0.560	[57]
117	Cd(NO_3_)_2_·4H_2_O	236.42	0.500	0.118	[41]
25	PH_3_Sn(CA)	757.50	0.006	0.005	[38]
26	*n*-BuSn(CA)	697.52	0.004	0.003	[38]
27	Ph_2_Sn(CA)_2_	1087.91	0.023	0.025	[38]
28	(*n*-Bu)_2_Sn(CA)_2_	1047.92	0.006	0.006	[38]
118	[Sn(2Am4DH)Cl_3_]	419.28	0.025	0.010	[58]
119	[Sn(2Am4Me)Cl_3_]	433.31	0.014	0.006	[58]
120	[Sn(2Am4Et)Cl_3_]	447.36	0.013	0.006	[58]
121	[Sn(2Am4Ph)Cl_3_]	495.40	0.002	0.001	[58]
122	[(n-Bu_2_Sn)_2_L]	816.11	0.032	0.039	[59]
123	MeSnCl(dact)	458.55	0.081	0.037	[60]
124	BuSnCl(dact)	500.62	0.133	0.061	[60]
125	PhSnCl(dact)	520.62	0.040	0.018	[60]
126	Ph_2_Sn(dact)	562.28	0.022	0.010	[60]
127	Bu_2_Sn(Acac)(4-MePCDT)	506.28	0.165	0.084	[61]

The LD_50_ value of Ni complex (**34**) with the Schiff base 3-((4-phenylthiazol-2-ylimino) methyl)-2-hydroxybenzoic acid (L) against brine shrimp was 117.4 μg/mL, while the corresponding value of free ligand was 254.7 μg/mL (Table 2) [40].

The toxicity of Ni complexes with formula [Ni_2_L^1^_2_(μ-1,1-N_3_)_2_(N_3_)_2_]·4H_2_O (**35**), and ([Ni_2_L^2^_2_(*μ*-1,1-N_3_)_2_(N_3_)_2_]·6H_2_O) (**36**) (H_2_L^1^Cl = (E)-*N,N,N*-trimethyl-2-oxo-2-(2-(1-(thiazol-2-yl)ethylidene)hydrazinyl)ethan-1-aminium chloride, H_2_L^2^Cl = (E)-*N,N,N*-trimethyl-2-oxo-2-(2-(1-(pyridin-2-yl)ethylidene)hydrazinyl)ethan-1-aminium chloride) exhibit LC_50_ 0.86 and 0.82 mM, respectively (Table 2). The positive control (K_2_Cr_2_O_7_) shows LD_50_ 0.077 mM [41].

The complexes of formulae [Ni(L^i^)_2_Cl_2_] (L^i^ = L^1^-L^6^) [L^1^ = N-(4,6-Dimethylpyrimidine-2-yl)-4-[furan-2ylmethylene)amino] benzene sulfonamide, L^2^ = 4-[(Furan-2-ylmethylene)amino]benzene sulfonamide, L^3^ = 4-{2-[(Furan-2-ylmethylene)amino]ethyl} benzenesulfonamide, L^4^ = 4-[(Furan-2-ylmethylene)amino]-N-(5-methylisoxazol3-yl)benzenesulfonamide, L^5^ = 4-[(5-Methylfuran-2-ylmethylene)amino]benzenesulfonamide, L^6^ = 4-{2-[(5-Methylfuran-2-ylmethylene)amino]ethyl} benzenesulfonamide] (**37–42**) were tested for theirs in vivo toxicity, indicating their LC_50_ values are higher than 1.18 mM, expect from Ni(L^6^)Cl_2_ with an LD_50_ value of 0.192 mM (Table 2) [42].

Nickel(II) complexes of 2,3-dihydroxybenzaldehyde N4-substituted thiosemicarbazone, (H_3_L^1^: R = H, H_3_L^2^: R = CH_3_, H_3_L^3^: R = C_6_H_5_ and H_3_L^4^: R = C_2_H_5_) (**43–50**) show a range of LD_50_ values between 0.059 to 0.096 mg/mL (Table 2) [43].

The LC_50_ value is 0.64 mM for Ni(BF_4_)_2_·6H_2_O (**51**) (Table 2) [41].

### Group 11 metals (Cu, Ag, Au) complexes

Copper*:* The in vivo toxicity of copper complex with amantadine (AdNH_2_), {[AdNH_3_^+^]·[CuCl_3_]^−^} (**52**), was examined through *A. salina* assay. The larvae were exposed to long range of concentrations. The LC_50_ (or LD_50_) value was determined at 0.428 mΜ (0.138 mg/mL) (Table 2) [44].

The complexes of formulae [Cu(L^i^)_2_Cl_2_] (**53–58**) (L^i^ = L^1^-L^6^) [L^1^ = N-(4,6-Dimethylpyrimidine-2-yl)-4-[furan-2ylmethylene)amino] benzene sulfonamide, L^2^ = 4-[(Furan-2-ylmethylene)amino]benzene sulfonamide, L^3^ = 4-{2-[(Furan-2-ylmethylene)amino]ethyl} benzene sulfonamide, L^4^ = 4-[(Furan-2-ylmethylene)amino]-N-(5-methylisoxazol3-yl)benzenesulfonamide, L^5^ = 4-[(5-Methylfuran-2-ylmethylene)amino]benzenesulfonamide, L^6^ = 4-{2-[(5-Methylfuran-2-ylmethylene)amino]ethyl} benzenesulfonamide] were tested in vivo toxicity. The LC_50_ values are in the range of 0.182 to higher than 1.5 mM (Table 2) [42].

The LC_50_ values of compounds with formulae Cu(L^i^–H)_2_(H_2_O)_2_ (**59–64**) (L^i^ L_1_ = N-(4,6-dimethylpyrimidin-2-yl)-4-[(2-hydroxynaphthalen-1-yl)methyleneamino]-benzenesulfonamide, L_2_ = *N*-(pyrimidin-2-yl)-4-[(2-hydroxynaphthalen-1yl)methyleneamino]-benzenesulfonamide, L_3_ = *N*-(3,4-dimethylisoxazol-5-yl)-4-[(2-hydroxynaphthalen-1-yl)methyleneamino]- benzenesulfonamide, L^4^ = *N*-(5-methylisoxazol-3-yl)-4-[(2-hydroxynaphthalen1-yl)methyleneamino]- benzene sulfonamide, L^5^ = *N*-(thiazol-2-yl)-4-[(2-hydroxynaphthalen-1yl)methyleneamino]- benzene sulfonamide, L^6^ = *N*-carbamimidoyl-4-[(2-hydroxynaphthalen-1yl)methyleneamino]- benzenesulfonamide) toward *A. salina* assay are in the range of 484 mM to higher than 1000 mM (Table 2) [45].

Complexes of formula Cu(L^x^-H)_2_(H_2_O)_2_ (**65–67**) [L^i^ = 4-[(2-hydroxynaphthalen-1-yl)methyleneamino] benzenesulfonamide, L^ii^ = 4-[{(2-hydroxynaphthalen-1-yl)methyleneamino}methyl] benzenesulfonamide and L^iii^ = 4-[2-{(2-hydroxynaphthalen-1-yl)methyleneamino} ethyl] benzenesulfonamide] were in vivo tested by *A. salina* assay. The range of LC_50_ values is between 490 and 676 mM (Table 2) [46].

The isonicotinoylhydrazide Schiff’s bases [L^1^ = *N*-(2-Furylmethylidene)nicotinohydrazide, L^2^ = *N*-(5-Methyl-2-furylmethylidene)nicotinohydrazide, L^3^ = *N*-(5-Nitro-2-furylmethylidene)nicotinohydrazide, L^4^ = *N*-(2-Thienylmethylidene)nicotinohydrazide, L^5^ = *N*-(5-Methyl-2-thienylmethylidene)nicotinohydrazide and L^6^ = *N*-(5-Nitro-2-thienylmethylidene) nicotinohydrazide] were used for the synthesis of Cu(II) complexes of formula [Cu(L^i^)_2_Cl_2_] (L^i^ = L^1^-L^6^) (**68–73**). The LD_50_ values lie between 0.354 to higher than 1 mg/mL (Table 2) [47].

*A. salina* larvae were incubated with 0.1 mg/mL of naphthoyl hydrazonoate copper complexes of formulae Cu(L^i^)_2_, (3-hydroxyl-2-naphthoylhydrazones containing pyrrole (HL^1^), furane (HL^2^) and thiophene (HL^3^) moieties) (**74–76**) for 24 h. The percentage of dead organisms upon their incubation with the samples **74–76** is 77.4, 92.8 and 43.1%, respectively (Table 2) [48].

The copper complexes [Cu(H2Am4DH)Cl_2_], [Cu(H2Am4Me)Cl_2_], [Cu(H2Am4Et)Cl_2_] and [Cu(2Am4Ph)Cl] (**77–80**) (H2Am4DH = 2-pyridineformamide thiosemicarbazone, H2Am4Me = *N*(4)-methyl-2-pyridineformamide thiosemicarbazone, H2Am4Et = N(4)-ethyl-2-pyridineformamide thiosemicarbazone, H2Am4P = *N*(4)-phenyl-2-pyr1idineformamide thiosemicarbazone were tested through *A. salina* assay. The LC_50_ values lie between 0.001 and 0.012 mM (Table 2) [49].

The toxicity of copper complexes [CuLCl](NO_3_), [CuLCl](ClO_4_) and [Cu_2_L_2_(*μ*-1,1-N_3_)_2_](ClO_4_)_2_), (**81–83**) (H_2_LCl = (E)-*N,N,N*-trimethyl-2-oxo-2-(2-(1-(pyridin-2-yl)ethylidene)hydrazinyl)ethan-1-aminium chloride), as well as the salts Cu(ClO_4_)_2_·6H_2_O and Cu(NO_3_)_2_·3H_2_O was tested against *A. salina* with a range of LC_50_ 0.46 to 1.54 mM (Table 2) [41]

The in vivo toxicity of the copper(II) complexes [CuCl_2_(INH)_2_]·H_2_O (**84**), [Cu(NCS)_2_(INH)_2_]·5H_2_O (**85**) and [Cu(NCO)_2_(INH)_2_]·4H_2_O (**86**) (INH = isoniazid) was tested against *A. salina*, The LD_50_ values were in the range of 0.008 to 0.244 mg/mL (Table 2) [50].

The LC_50_ values of copper complexes of ONNO, NNNO, ONNS & NNNS donor tetra-dentate Schiff bases (L_1_-L_12_) and formulae [Cu(L^i^)(H_2_O)Cl] (**87–98**) ((L^1^ = 2-[(2-{[(2-furylmethylene]amino}phenyl)imino]methyl}-phenol, L^2^ = 2-[(2-{[(2-Thienylmethylene]amino}phenyl)imino]-methyl}phenol, L^3^ = 2-[(2-{[(1H-pyrrol-2-ylmethylene] amino} phenyl)-imino] methyl}phenol, L^4^ = 2-[(2-{[(2-Furylmethylene] amino}phenyl)imino]-methyl}thienyl, L^5^ = 2-{[2-(2-Furylmethylene]amino}phenyl)imino]-methyl}pyrrol, L^6^ = 2-{[2-(2-Thienyllmethylene]amino}phenyl)imino]-methyl}pyrrol, L^7^ = 2-{[2-(2-Furyllmethylene] amino}ethyl)imino]methyl}-phenol, L^8^ = 2-{[2-(2-Thienyllmethylene] amino}ethyl)imino]methyl}-phenol, L^9^ = 2-{[2-(2-Pyrollylmethylene]amino}ethyl)imino]methyl}-phenol, L^10^ = 2-[(2-{[(2-Furylmethylene]amino}ethyl)imino]methyl}-thienyl, L^11^ = 2-{[2-(2-Furylmethylene] amino}ethyl)imino]methyl}-pyrrol, L^12^ = 2-{[2-(2-Thienyllmethylene] amino}ethyl)imino]methyl}-pyrrol) are between 0.87 to higher than 2.9 mM (Table 2) [51].

Copper salts: The LC_50_ value is 0.24 mM for Cu(NO_3_)_2_·3H_2_O (**99**) (Table 2) [41]. Moreover, the LC_50_ value of CuCl_2_·2H_2_O (**100**) was 7.0 μM [49]. The LC_50_ values of Cu(ClO_4_)_2_·6H_2_O (**101**) is 0.28 mM (Table 2) [41].

Silver(I): The combination of penicillin G (PenH) with silver(I) ions resulted in the formation of a new metallodrug with the formula ([Ag(pen)(CH_3_OH)]_2_) (**102**). Its toxicity was evaluated through *A. salina* assay at a range of concentration 0.04 to 1.05 mΜ. The LC_50_ was determined at 0.532 mM (or 0.504 mg/ml) (Table 2) [52].

The extract from oregano leaves (ORLE) was used for the synthesis of silver nanoparticles, AgNPs(ORLE) (**103**). The tested concentrations were in the range of 150 to 300 mg/mL. The LC_50_ was determined 217.8 mg/mL (Table 2) [53].

### Group 12 metals (Zn, Cd, Hg) complexes

Zinc*:* Two zinc complexes [Zn(valp)_2_phen(H_2_O)] (**104**) and Zn(valp)_2_(bipy) (**105**) (valp = valproic acid, phen = 1,10-phenathroline, bipy = 2,2-bipyridine) show LD_50_ value against *A salina* 0.078 and 0.409 mg/mL respectively (Table 2) [54].

The LC_50_ value of compound Zn(INH)_2_](ClO_4_)_2_·6H_2_O (**106**) (INH = isoniazid) was calculated at 268 μM (Table 2) [55].

The LC_50_ values of zinc complexes, [ZnL^1^(NCS)_2_]·2H_2_O (**107**) and [ZnL^2^(NCS)_2_]·0.5MeOH (**108**) (HL^1^Cl ligand = (E)-*N,N,N*-trimethyl-2-oxo-2-(2-(1-(thiazol-2-yl)ethylidene)hydrazinyl)ethan-1-aminium chloride, HL^2^Cl = (E)-*N,N,N*-trimethyl-2-oxo-2-(2-(1-(pyridin-2-yl)ethylidene)hydrazinyl)ethan-1-aminium chloride, NCS = N-Chlorosuccinimide), are calculated at 1.27 and 0.98 mM, respectively (Table 2) [41].

The LD_50_ of zinc salts, Zn(BF_4_)_2_·6H_2_O (**109**) and Zn(OAc)_2_·2H_2_O (**110**), exhibited a range of 0.88 to 1.18 mM (Table 2) [41].

Cadmium complexes of thiophene-2,3-dicarboxaldehyde bis(thiosemicarbazone) (2,3BTSTCH_2_) with formulae [CdCl_2_(2,3BTSTCH_2_)] (**111**) and [CdBr_2_(2,3BTSTCH_2_)] (**112**) were assessed through *A. salina* test. The LC_50_ (or LD_50_) values were 0.3 mM (or 0.115 mg/mL) (**111**) and 0.24 (or 0.132 mg/mL) (**112**) mM, respectively (Table 2) [56].

The LC_50_ value of the complex CdHL^3^(NCS)_3_ (**113**) (HL^3^Cl = (E)-*N,N,N*-trimethyl-2-oxo-2-(2-(1-(pyridin-2-yl)ethylidene)hydrazinyl)ethan-1-aminium chloride, NCS = *N*-Chlorosuccinimide) is 0.53 mM (Table 2) [41].

Cd complexes with derivatives of 2-acetylpyridine ethyl hydrazinoacetate hydrochloride (aphaOEt) or 2,6-diacetylpyridine ethyl hydrazinoacetate hydrochloride (dapha(OEt)_2_, formulae CdCl_2_(aphaOEt)(DMF) (**114**) and [CdCl_2_(dapha(OEt)_2_)]·1.5H_2_O (**115**), show LC_50_ values 3.30 and 1.39 mM, respectively (Table 2) [57].

The LC_50_ value of CdCl_2_ (**116**) is 3.03 mM [57] and 0.50 mM for Cd(NO_3_)_2_·4H_2_O (**117**) (Table 2) [41].

### Group 14 metals (Sn, Pb) complexes

Organotins*:* Organotin compounds derived from cholic acid (CAH) R_3_Sn(CA) [R = Ph- (**25**), n-Bu- (**26**)] and R_2_Sn(CA)_2_ [R = Ph- (**27**) and n-Bu- (**28**)] were evaluated for their in vivo toward *A. cepa* and were also studied using *A. salina*. The range of LC_50_ values are between 3.9 and 23.3 μΜ (Τable 2) [38].

Tin(IV) complexes [Sn(2Am4DH)Cl_3_] (**118**), [Sn(2Am4Me)Cl_3_] (**119**), [Sn(2Am4Et)Cl_3_] (**120**) and [Sn(2Am4Ph)Cl_3_] (**121**) (H2Am4DH = 2-pyridineformamide thiosemicarbazone, H2Am4Me = *N*(4)-methyl 2-pyridineformamide thiosemicarbazone, H2Am4Me = *N*(4)-methyl 2-pyridineformamide thiosemicarbazone, H2Am4Et = *N*(4)-ethyl 2-pyridineformamide thiosemicarbazone, H2Am4Ph = *N*(4)-phenyl-pyridineformamide thiosemicarbazone) presented LC_50_ values between 1.6 and 25.5 μM (Table 2) [58].

The compound [(*n*-Bu_2_Sn)_2_L] (**122**) (L = *N*^1'^,*N*^4'^-bis(2-oxidobenzylidene)succinohydrazide) presented an LD_50_ value of 32.11 μg/mL (Table 2) [59].

Tin complexes MeSnCl(dact) (**123**), BuSnCl(dact) (**124**), PhSnCl(dact) (**125**), Ph_2_Sn(dact) (**126**) (H_2_dact = 2-hydroxyacetophenone-*N*(4)-cyclohexylthiosemicarbazone) exhibited potential cytotoxic activity against *A. salina*, as their LC_50_ values were up to 61.20 ppm or up to 133.5 μΜ (Table 2) [60].

The diorganotin(IV) derivative of 4-methyl-1-piperidinecarbodithioic acid (4-MePCDTA) of formula Bu_2_Sn(Acac)(4MePCDT) (**127**) was also tested via *A. salina* assay a LD_50_ value of 83.7 μg/mL (Table 2) [61].

## Conclusion

The biological effects of metal ions and their compounds in the living organisms (*A cepa* and *A salina*) are reviewed here with the aim on the development of in vivo toxicity models for the evaluation of their genotoxicity and toxicity. To accomplish this goal, their microscopic parameters (such as MI and CA) as well as their macroscopic ones (root length) were reviewed and compared, and the LC_50_ or LD_50_ values are summarized.

The study revealed that some CAs are usually observed after the treatment with a metal ion [16, 17, 20, 27–29, 37, 38, 62] (Table 3). However, a specific abnormality of the chromosomes could not be linked with the presence of a particular metal ion, since different metal ions may promote the appearance of the similar result. In agreement to this, Leme et al. [14] reported previously that the grouping of metal ions regarding their cytological effects is not possible.

**Table 3:** Summary of the most common CAs induced by a specific metal

Metal	Most common CAs	Refs.
Ag	Chromosome adherences, chromosome losses, single bridges and fragments	[16, 20, 27–29]
Sb	Stickiness, bridges and vagrant chromosomes	[17, 38]
Cd	Chromosomal bridge, break, stickiness, clumping, c-mitosis, stickiness	[37]
Ni	C-mitosis	[62]
Hg	Stickiness	[62]

Moreover, the value % Mitotic Index Alteration (%MIA(C)) was introduced to overcome the quality of control water used as well as the variety of *A. cepa* bulb types. A substance could be considered as non-toxic, if it promotes the death of < 30% of the cells (viability ≥ 70%) (ISO 10993-5:2009) [21, 22]. This classification is extended within this work to categorize any agent that caused %MIA(C) ≤ 70% as a potent genotoxic one, with the rests to be considered as a non-genotoxic.

No conclusion can be withdrawn for the time scale (24 or 48 h) of the effect since no sufficient data are available (Table 1). On the contrary, Jaishankar et al. [63], have reported that metal ion toxicity depends not only on its dosage but on the duration of this exposure as well [63].

**Fig. 3:**
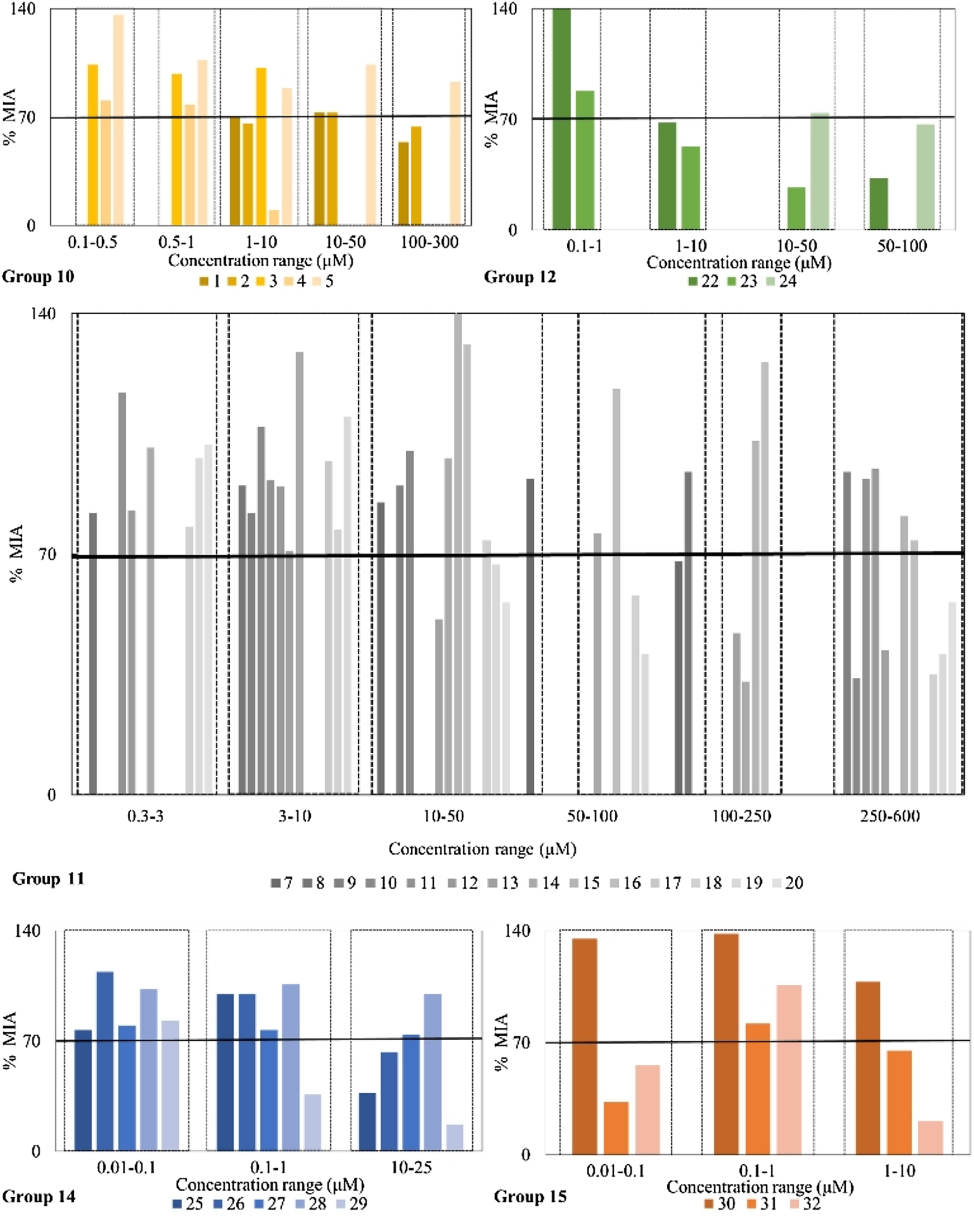
%MIA in *A. cepa* root cells induced by exposure to different concentrations of groups 10, 11, 12, 14 and 16

Among the metal ions and their compounds of the group of elements studied here, those of 12 show %MIA ≤ 70 against *A cepa* at lower concentration (1–10 μΜ), since they affecting strongest the mitosis of the bulb (Table 1, Fig. 3). However, lacking a large number of samples that would lead to reliable conclusions for the elements of all groups studied the very low toxicity of silver and its compounds can be suggested (%MIA ≤ 70 at 250–600 μΜ) (Fig. 3).

Comparing the % MIA of silver(I) complexes with various ligands, differences in genotoxicity are observed (Fig. 3). Therefore, the presence of the ligand affects the genotoxicity of the metal ion, as it alters its environment [64]. This is expected since different chemical environment of the metal ion influences the lipophilicity of the complex and, as a consequence, its ability to permeate the cell membrane [65]. Thus, different ligands lead to different absorption and uptake levels in different organs or cell organelles [66]. These differences result in a wide range of toxicity observed. Moreover, the precursor of the gold complexes [Au(tpp)Cl] [20] does not affect the mitotic index up to the concentration of 30 μΜ. In the case of the tin and antimony complexes, their genotoxicity is induced at the concentrations of 10 and 0.01 μΜ, respectively.

In the case of *Artemia salina* assay, the mean of LC_50_ values of the complexes is between 0.04 and 126 mM. The most potent toxic compounds seem to be the tin compounds (LC_50_^mean^ = 0.04 mM, count = 14), while the less toxic seems to be the copper complexes (LC_50_^mean^ = 126, count = 32). Generally, the toxicity order is Cu < Zn < Cd < Ni < Sn (with LC_50_^mean^ 126 (Cu), 39 (Zn), 1.3 (Zn), 0.29 (Ni) and 0.04 (Sn) mM.

In conclusion, two biological assays, namely *Allium cepa* and *Artemia salina*, were reviewed regarding the toxicity risk assessment of metal ions. The findings highlight the effect of the metal ions and their complexes in the biological systems, such as plants, aquatic organisms and hence humans. Their toxicity is in high relevance with their concentration. Considering that humankind is continuously dependent on surface waters the contribution of the environmental biological inorganic chemistry toward the refinement of the environment can be of great importance, and it initiates a new era in the field of environmental chemistry and biological sciences.

## Abbreviations

%MIA: % Mitotic Index Alteration
2,3BTSTCH_2_: Thiophene-2,3-dicarboxaldehyde bis(thiosemicarbazone)
AdNH_2_: Amantadine
aphaOEt: 2-Acetylpyridine ethyl hydrazinoacetate hydrochloride
bipy: 2,2-Bipyridine
BzimetTSCH: 1-(1*H*-Benzimidazol-2-yl)ethan-1-one thiosemicarbazone
CA: Chromosomal abnormalities
CAH: Cholic acid
CIPH: Ciprofloxacin
dapha(OEt)_2_: 2,6-Diacetylpyridine ethyl hydrazinoacetate hydrochloride
FAO: Food and Agriculture Organization of the United Nations
GlyH: Glycine
H2Am4DH: 2-Pyridineformamide thiosemicarbazone
H2Am4Et: *N*(4)-Ethyl-2-pyridineformamide thiosemicarbazone
H2Am4Me: *N*(4)-Methyl-2-pyridineformamide thiosemicarbazone
H2Am4P: *N*(4)-Phenyl-2-pyr1idineformamide thiosemicarbazone
H_2_mna: 2-Mercapto-nicotinic acid
INH: Isoniazid
LC_50_: Lethal Concentration (mM) that eliminates the 50% of the nauplii
LD_50_: Lethal Dose (mg/mL) that eliminates the 50% of the nauplii
Me_2_DTC: Dimethyldithiocarbomate
MI: Mitotic index
MMI: 2-Mercapto-1-methyl-imidazole
MN: Micronucleus
NA: Nuclear abnormalities
NCS: *N*-Chlorosuccinimide
NMP: *N*-Methyl pyrrolidone
ORLE: Extract from oregano leaves
PenH: Penicillin G
phen: 1,10-Phenathroline
salH_2_: Salicylic acid
SCP: Sulfachloropyridazine
SDM: Sulfadimetoxine
SMX: Sulfamoxole
TPP: Triphenylphophine
valp: Valproic acid
WHO: World Health Organization
